# Characterization of Corneal Defects in ATG7-Deficient Mice

**DOI:** 10.3390/ijms26209989

**Published:** 2025-10-14

**Authors:** Thomas Volatier, Andreas Mourier, Johanna Mann, Berbang Meshko, Karina Hadrian, Claus Cursiefen, Maria Notara

**Affiliations:** 1 Department of Ophthalmology, Faculty of Medicine and University Hospital Cologne, University of Cologne, 50923 Köln, Germany; 2 Center for Molecular Medicine Cologne (CMMC), Faculty of Medicine and University Hospital Cologne, University of Cologne, 50923 Köln, Germany; 3 Cluster of Excellence for Aging Research (CECAD), Faculty of Medicine and University Hospital Cologne, University of Cologne, 50923 Köln, Germany

**Keywords:** cornea, autophagy, corneal epithelium, ATG7, corneal homeostasis

## Abstract

Regulated proteolysis via autophagy is essential for cellular homeostasis, yet the specific role of autophagy-related gene 7 (ATG7) in corneal epithelial maintenance remains unclear. Using a conditional knockout mouse model (*Atg*7^f/f^ K14Cre^+/−^), we investigated the impact of ATG7 deficiency on corneal epithelial autophagy, morphology, and vascular dynamics. Loss of ATG7 disrupted autophagosome formation, evidenced by increased LC3B expression but reduced LC3B-positive puncta and absence of autophagosomes ultrastructurally. Although gross corneal morphology was preserved, ATG7 deficiency led to thickened epithelium and increased peripheral lymphatic vessel sprouting, indicating a pro-inflammatory and pro-lymphangiogenic microenvironment. Proteomic analysis revealed upregulation of RAB8, TM9S3, and RETR3, suggesting activation of compensatory pathways such as exophagy, reticulophagy, and Golgiphagy. Inflammatory and angiogenic components were downregulated, suggesting a moderate loss of inhibitory capacity based on the lymphatic phenotypes observed. At the same time, while these two compensatory changes occur, other proteins that positively regulate lysosome formation are reduced, resulting in a phenotype linked to deficient autophagy. These findings demonstrate that ATG7-mediated autophagy maintains corneal epithelial homeostasis and immune privilege, with implications for understanding corneal inflammation and lymphangiogenesis in ocular surface diseases.

## 1. Introduction

Regulated proteolysis is essential for maintaining cellular homeostasis. This process involves the enzymatic degradation of proteins into peptides and amino acids that can be recycled for the synthesis of new proteins. Cells utilize two primary pathways to accomplish regulated protein degradation: the ubiquitin-proteasome system (UPS) and the lysosomal proteolysis pathway [1]. The UPS primarily degrades short-lived and misfolded proteins in the cytoplasm and nucleus. The lysosomal system degrades membrane-bound, extracellular, and intracellular components delivered via endocytosis or autophagy [2].

Autophagy, a central component of the lysosomal degradation system, encompasses three main types: microautophagy, chaperone-mediated autophagy, and macroautophagy. Of these, macroautophagy (hereafter simply referred to as autophagy) is the most thoroughly characterized. Autophagy is a highly conserved, intracellular degradation process involving the sequestration of cytoplasmic components within double-membrane autophagosomes, which subsequently fuse with lysosomes for degradation [3,4]. Autophagy plays a pivotal role in cellular quality control by eliminating damaged organelles, misfolded or aggregated proteins, and invading pathogens. It also enables cells to adapt to metabolic stress and contributes to immune responses, development, and tissue remodeling [5].

A critical enzyme in the autophagic machinery is Autophagy-related gene 7 (ATG7), which acts as an E1-like activating enzyme necessary for the lipidation of LC3 (microtubule-associated protein 1 light chain 3) and the conjugation of ATG12 to ATG5, both of which are essential steps for autophagosome formation [6]. Genetic deletion of Atg7 in mice results in the complete blockade of autophagy, leading to intracellular accumulation of ubiquitinated protein aggregates, defective organelle turnover, and heightened cellular stress responses [7,8]. These phenotypes underscore the indispensable role of ATG7 in the maintenance of cellular homeostasis. This cellular homeostasis in the epithelial cell is a part of the greater corneal homeostasis, where the healthy epithelial cells undergo a regulated division, differentiation, and migration to match the rate at which superficial cells slough off from the cornea [9]. A disruption of intracellular autophagy can magnify to tissue-wide homeostatic imbalance and vulnerability.

Although systemic ATG7-deficient mouse models have been instrumental in defining the global consequences of autophagy loss, including neurodegeneration, hepatomegaly, and perinatal lethality, tissue-specific roles of ATG7 remain incompletely understood [9]. Conditional knockout models such as the K14-Cre; *Atg7*^f/f^ mouse, which deletes Atg7 in keratin 14-expressing epithelial tissues, have revealed important insights. In the skin, ATG7-deficient keratinocytes exhibit disrupted autophagic flux, accumulation of p62-positive aggregates, and increased sensitivity to UVB-induced apoptosis, despite preserving gross barrier function [10,11].

Given the parallels between skin and corneal epithelium, both being stratified, keratinized epithelial barriers frequently exposed to environmental insults, the role of ATG7-mediated autophagy in the cornea is of significant interest. The corneal epithelium is constantly exposed to ultraviolet (UV) radiation, oxidative stress, and mechanical insult, necessitating robust mechanisms for cellular turnover, stress resistance, and barrier integrity [12,13]. Autophagy in the cornea has been implicated in wound healing and modulation of inflammatory responses [14,15,16]. Dysregulation of protein recycling in the corneal epithelium occurs in corneal diseases such as keratoconus and can contribute to further epithelial dystrophies and degenerative diseases [17]. Autophagy also mitigates inflammation by reducing the presence of inflammatory factors [18].

Despite the central role of ATG7 in autophagy and emerging evidence for autophagic function in corneal biology, the specific contribution of ATG7 to corneal epithelial homeostasis remains poorly defined. Given that ATG7-deficient epidermal models exhibit exacerbated UV damage and age-associated defects, it is plausible that loss of autophagy in the corneal epithelium may similarly compromise tissue resilience and clarity.

Recent studies have further elucidated the protective significance of autophagy in skin under stress. For instance, myofibroblast-specific ATG7-deficiency exacerbates radiation-induced skin injury and fibrosis, highlighting how epithelial and stromal autophagy contribute to tissue resilience following environmental insults [19]. These findings are complemented by data demonstrating that autophagy in epidermal cells supports cornification, barrier integrity, and immune modulation. Keratinocytes lacking ATG7 display impaired nuclear removal, aberrant lipid composition, and hampered differentiation [20]. Together, these results reinforce that autophagy not only enables routine epithelial maintenance but also serves as a defensive mechanism during acute and chronic stress in the skin.

Additionally, in corneal alkali burn models, downregulation of ATG7, ATG5, and Beclin-1 correlates with pathological neovascularization and inflammation, suggesting that autophagy mediates wound repair and suppresses maladaptive tissue remodeling [21]. Together, these cornea-specific findings underscore autophagy’s role in ocular epithelial defense.

Pathological neovascularization of the cornea often leads to progressive opacification. This neovascularization can be caused by infections, inflammation, hypoxia (such as from prolonged contact lens wear), trauma or surgery, chemical burns, limbal stem cell deficiency, and autoimmune diseases, all of which disrupt the cornea’s natural anti-angiogenic balance and promote abnormal blood vessel growth [22,23,24,25].

Autophagy plays a complex, context-dependent role in regulating neovascularization in tissues such as the cornea and skin. In the cornea, studies in mouse alkali burn models and human endothelial cells show that impaired autophagy promotes inflammatory macrophage activation and pathological corneal neovascularization, while pharmacological activation of autophagy reduces vessel growth [21]. Conversely, in skin, autophagy appears to facilitate angiogenesis in response to injury or mechanical stress. For example, keratinocyte-specific deletion of the autophagy gene Atg7 in mice reduces UVB-induced blood and lymphatic vessel formation, indicating that autophagy promotes angiogenesis under UV stress [26]. Activation of autophagy enhances angiogenesis during mechanical skin expansion in mice [27]. These findings suggest that while autophagy suppresses pathological neovascularization in the normally avascular cornea by modulating inflammation, it supports physiological angiogenesis in skin during tissue repair.

Neovascularization increases the risk of inflammation and immune cell infiltration, potentially compromising corneal immune privilege and increasing the likelihood of graft rejection after transplantation [28]. Therefore, maintaining corneal avascularity is critical for preserving its optical clarity and function [29].

In this study, we utilize a conditional knockout mouse model (*Atg7*^f/f^ K14Cre^+/−^) to cause an ATG7 deficiency in the corneal epithelium. We characterize the impact of ATG7 loss on autophagy, epithelial morphology, vascularization, and inflammation. The results of this investigation may provide insight into potential therapeutic targets for ocular surface diseases associated with epithelial dysfunction and impaired stress tolerance.

## 2. Results

### 2.1. ATG7 Deficiency in the Corneal K14 Compartment

**Figure 1:**
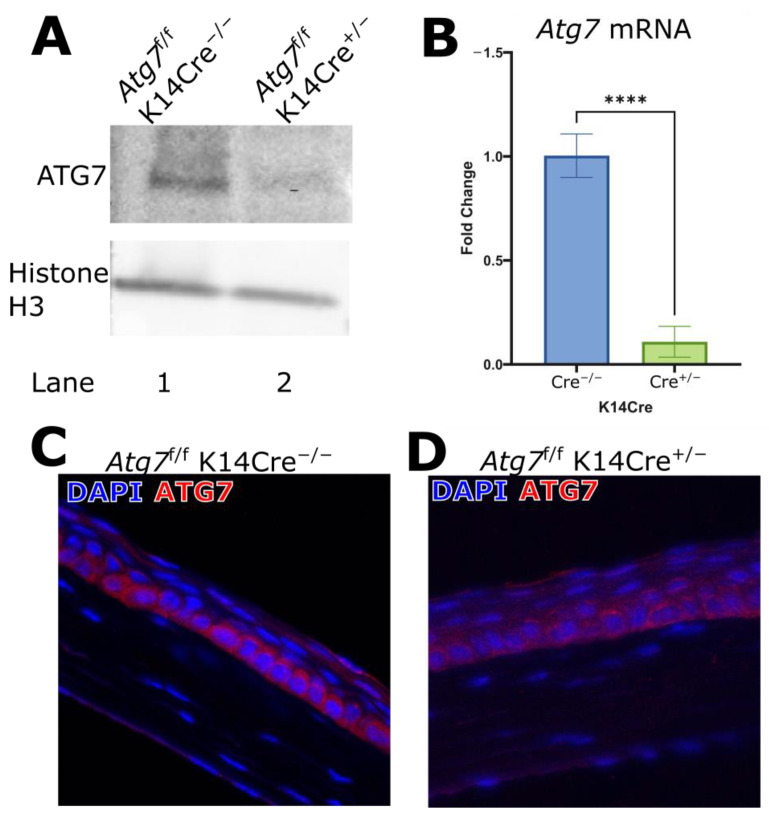
(**A**) Western blot of Atg7 in corneal epithelium lysates. The lysates of mouse corneal epithelium of the indicated genotypes were immunoblotted with ATG7 and Histone H3 (*n* = 4). (**B**) Quantitative RT-PCR of the mouse whole cornea to measure Atg7 expression; HPRT was used as a housekeeping gene (*n* = 6). (**C**,**D**) ATG7 deficiency is shown in the basal epithelial layer of the mouse central cornea. (**** indicates *p* ≤ 0.0001).

To investigate the autophagy-deficient *Atg7*mutant mouse model, the absence of ATG7 protein and *Atg7* mRNA had to be confirmed first. Importantly, mice homozygous for the floxed *Atg7* allele but lacking Cre (*Atg*7^f/f^) were viable, fertile, and displayed no overt abnormalities, as was previously described by the group that established the *Atg*7^f/f^ K14Cre^+/−^ [10]. Western blot analysis of corneal epithelial lysates shows a reduction in ATG7 protein levels in *Atg*7^f/f^ K14Cre^+/−^ mice compared to controls (Figure 1A), indicating depletion of ATG7 in the epithelium. In parallel, quantitative RT-PCR analysis demonstrated a significant reduction in ATG7 mRNA expression in the corneal epithelium of mutant mice, validating the loss of ATG7 at the transcriptional level (Figure 1B). Immunofluorescence staining of corneal tissue sections further confirmed a decrease in ATG7 protein, specifically within the basal layer of the corneal epithelium (Figure 1C,D), consistent with K14 promoter activity.

### 2.2. Autophagosome Formation in ATG7-Deficient Corneal Epithelium

**Figure 2:**
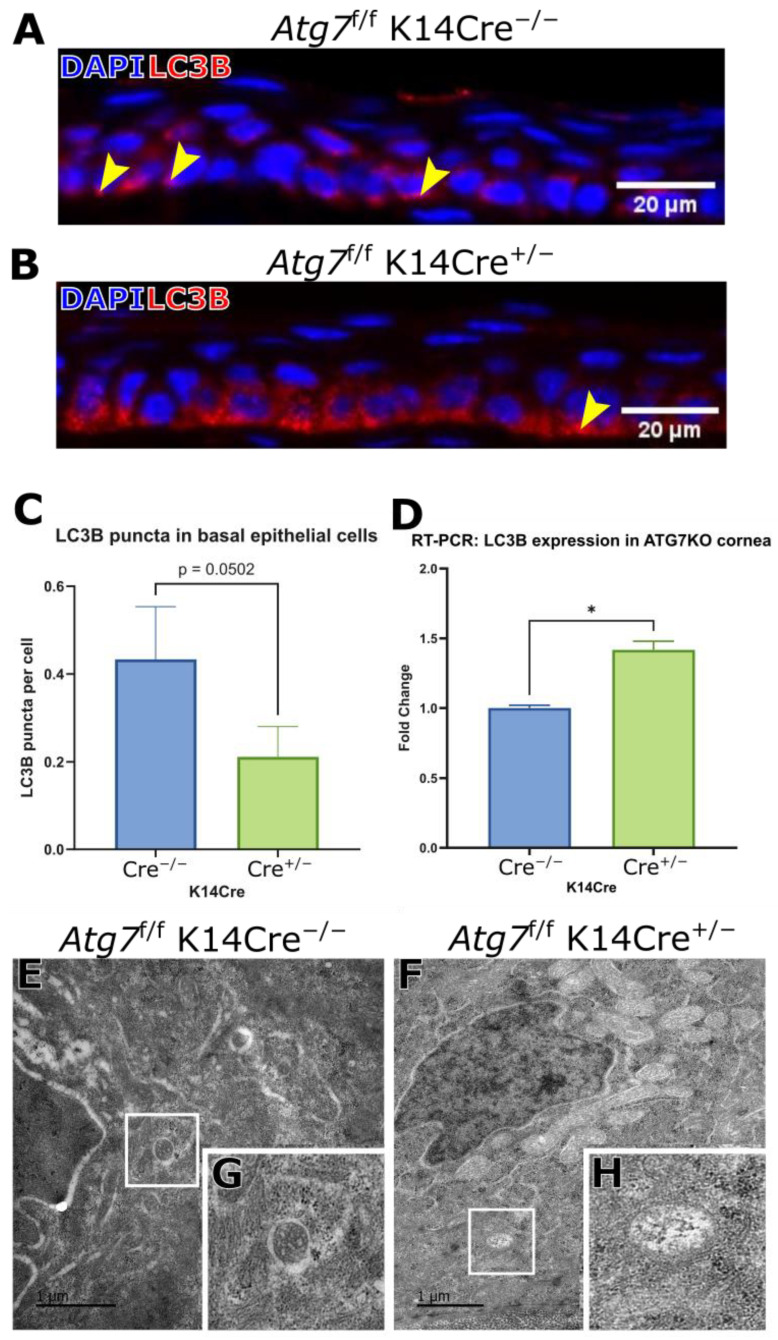
(**A**,**B**) show the increased distribution of LC3B in the basal corneal epithelium of ATG7-deficient mice. Indicated with yellow markers are puncta of LC3B. The number of puncta per basal layer cells is counted in (**C**), showing a significant reduction in the ATG7-deficient animals (*n* = 6). (**D**) RT-PCR analysis shows an increase in LC3B expression in the ATG7-deficient mice (*n* = 6). TEM images of (**E**) basal epithelial cell from the central epithelium of an *Atg7*^f/f^ K14Cre^−/−^ mouse cornea. (**F**) Basal epithelial cell from the central epithelium of an *Atg7*^f/f^ K14Cre^+/−^ mouse cornea. (**G**) Highlighted autophagosome with internal cargo. (**H**) Highlighted autophagosome with little to no internal cargo. (* indicates *p* ≤ 0.05).

In ATG7-deficient corneal epithelium, LC3B expression levels were significantly increased relative to controls (Figure 2A,B); however, LC3B-positive puncta were notably reduced in the basal epithelial layer (Figure 2C). This pattern is consistent with impaired autophagosome formation, as ATG7 is required for LC3B lipidation and autophagosome membrane expansion. Expression of LC3B, as measured by mRNA, was increased in the ATG7-deficient cornea (Figure 2D).

Transmission electron microscopy (TEM) of ATG7-deficient corneal epithelium revealed a change in autophagosome-like structures (Figure 2F,H), in contrast to control tissue, where characteristic double-membrane vesicles were occasionally observed (Figure 2E,G). Autophagosomes were identified by their double membrane and content; they were discriminated from mitochondria by excluding cristae-containing structures. The presence of content within was based on the darkness of the intra-organelle cargo, with whiter intra-organelle space being associated with little to no cargo [8].

### 2.3. Increased Corneal Epithelial Thickness of ATG7-Deficient Mouse Cornea

**Figure 3:**
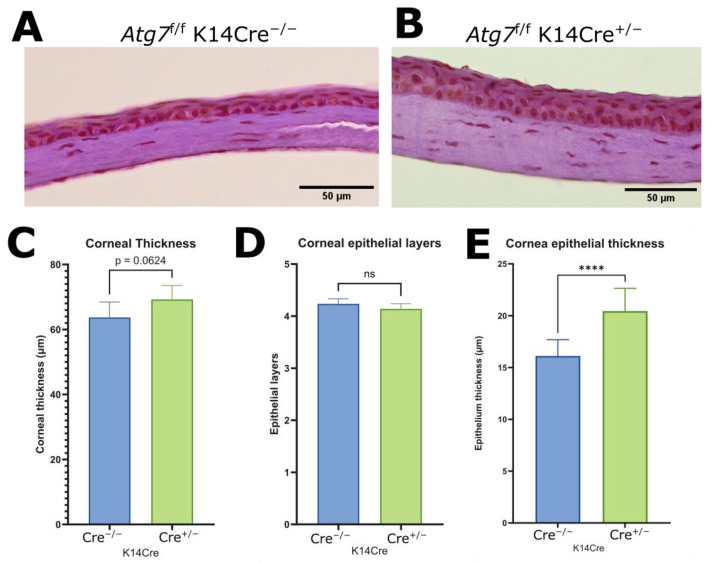
Hematoxylin and Eosin stain of control mouse cornea section (**A**) and ATG7-deficient mouse cornea section (**B**). Measurements of corneal thickness (**C**) (*n* = 6) and corneal epithelial layer count (**D**) (*n* = 6) showed no difference. Measurement of epithelial thickness showed increased epithelial thickness in the ATG7-deficient central cornea (**E**) (*n* = 11). (**** indicates *p* ≤ 0.0001, ns indicates *p* > 0.05).

Histological analysis revealed that, while overall corneal thickness was not increased (Figure 3A–C), a significant increase in central corneal epithelial thickness was observed in ATG7-deficient mice compared to controls (Figure 3E). Despite the thickening, the number of epithelial cell layers remained unchanged (Figure 3D), suggesting that the increased thickness is due to basal layer cellular hypertrophy or altered cell morphology rather than increased proliferation or stratification.

### 2.4. Corneal Vasculature Features of ATG7-Deficient Mouse Cornea

**Figure 4:**
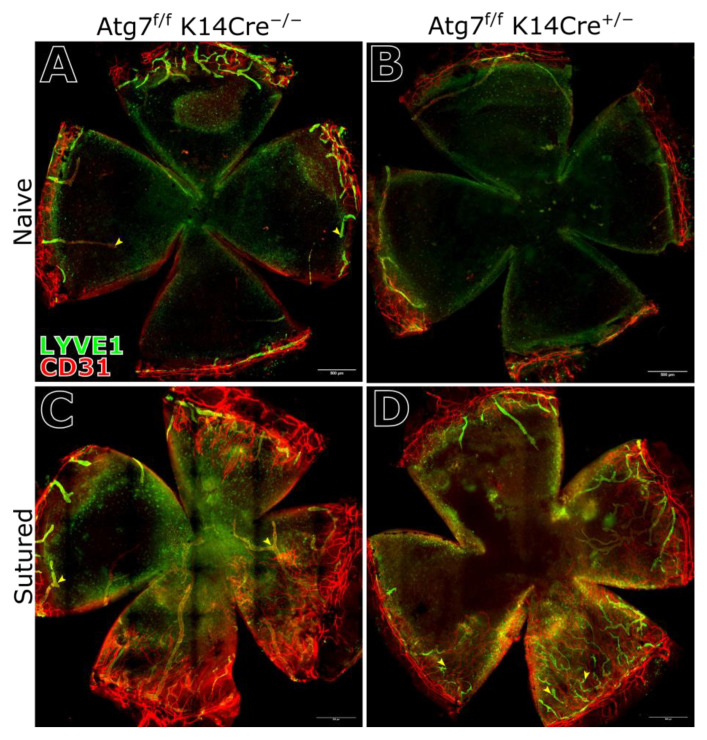
Corneal vasculature, blood and lymphatic vessels, labeled with CD31 and LYVE1, respectively. Naïve, undamaged cornea of control mice (**A**) and ATG7-deficient mice (**B**), where both show an avascular corneal center with a peripheral ring of vessels at the limbus. Corneas with sutures placed into the stroma for 2 weeks showed vessel invasion of the previously avascular corneal center in the cornea of control mice (**C**) and ATG7-deficient mice (**D**). Vessel features are also indicated with yellow arrows in (**A**) endpoints, in (**C**) branches, and in (**D**) sprouts.

**Figure 5:**
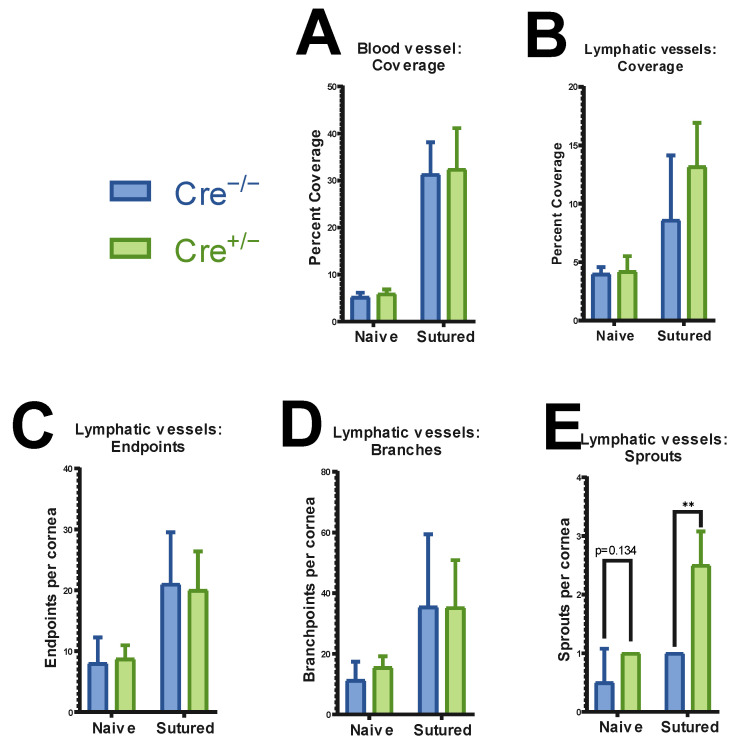
Morphometric analysis of corneal vasculature in naïve (*n* = 18) and sutured (*n* = 8) corneas of ATG7-deficient animals and controls. Vessel coverage showed no difference when comparing mutant and control mice, not in blood (**A**) or lymphatic (**B**) vascular coverage. Closer analysis of lymphatic vessel features showed no differences in the number of endpoints (**C**) or branches (**D**). A significant increase in the number of sprouts was observed in the sutured cornea of ATG7KO mice (**E**). (** indicates *p* ≤ 0.01).

Quantification of corneal vasculature of both naïve and sutured mice revealed no significant difference in overall blood vessel or lymphatic vessel coverage between ATG7-deficient and control mice. While suture placement did increase vessel coverage (Figure 4A–D), there was no difference in the vascularization of the cornea of ATG7-deficient compared to the control (Figure 5A,B). However, LYVE1 immunostaining showed a small but statistically significant increase in the number of lymphatic vessel sprouts in the peripheral cornea of ATG7-deficient mice (Figure 5C–E). This suggests that while gross vascular patterning remains unaffected, ATG7 loss has slight pro-lymphangiogenic activity.

### 2.5. Proteomic Analysis Reveals Compensatory Mechanism for Autophagy-Deficiency

**Figure 6:**
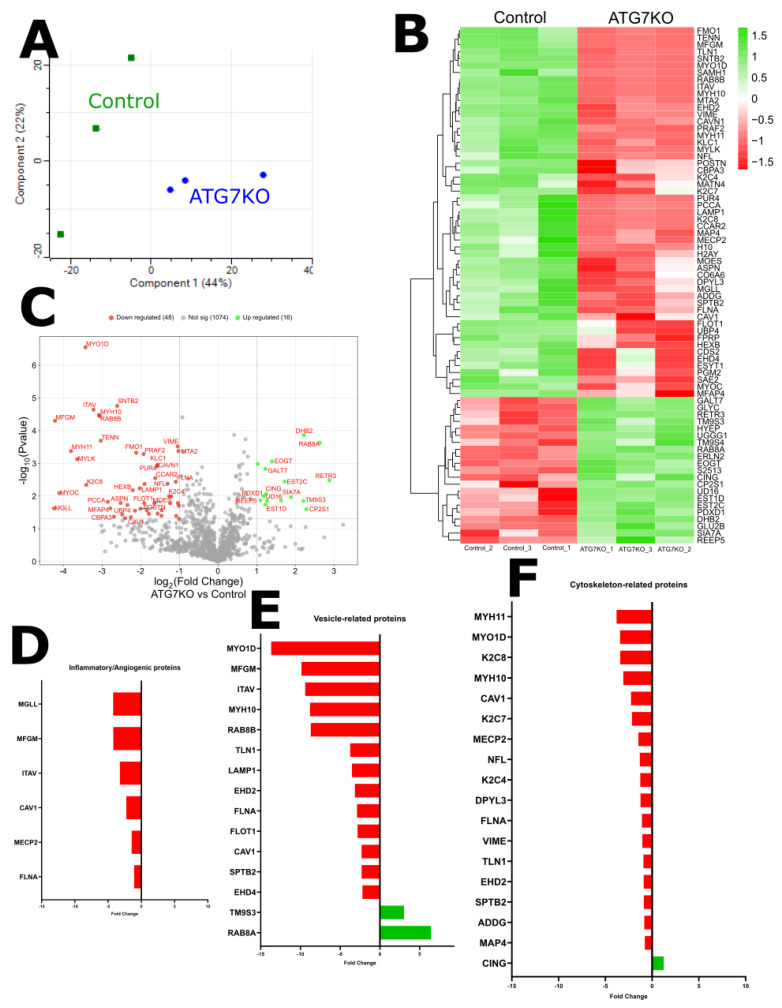
(**A**) PCA graph of ATG7KO and Control mice used for proteomics analysis (*n* = 3). (**B**) Heat map and (**C**) Volcano plot illustrating differentially expressed proteins in ATG7-deficient mice and control mice. Bar charts of differentially expressed proteins classed by groups: Inflammatory/Angiogenic (**D**), Vesicle-related (**E**), and Cytoskeleton-related (**F**). In all graphs, red indicates a reduction and green indicates an increase.

Proteomic profiling of whole corneal tissue from ATG7-deficient mice and control mice shows that the two genotypes exhibit a noteworthy degree of distance (Figure 6A). Given the relevance of autophagy as a biological process of interest, two components of non-ATG7-related autophagy were identified as having the highest enrichment in the ATG7-deficient cornea: RETR3 and RAB8A (Figure 6B,C). Associated processes of autophagy, like lysosomal degradation pathways, also seem affected. In our proteomic analysis, we see a reduction in LAMP1 quantity within the cornea of ATG7-deficient animals (Figure 6B,C).

A process not typically linked to autophagy, cytoskeletal arrangement, seemed most affected according to the proteomic panel (Figure 6F). Proteins like Talin-1, responsible for cell adhesion complex formation and linkage to actin; MYO1D and MYH11, both myosins, parts of the actomyosin cytoskeleton; and K2C4, K2C7, and K2C8, keratins involved in cytoskeleton assembly (Figure 6F). Several vesicle-related and transport-related proteins like CAV1, LAMP1, EHD2, EHD4, FLOT1, RAB8B, and MYO1D are reduced in autophagy-deficient mouse corneas (Figure 6E). Angiogenesis-related proteins such as MFGE8, integrin αV (ITGAV), and MECP2 are reduced in autophagy-deficient mouse corneas (Figure 6D).

**Figure 7:**
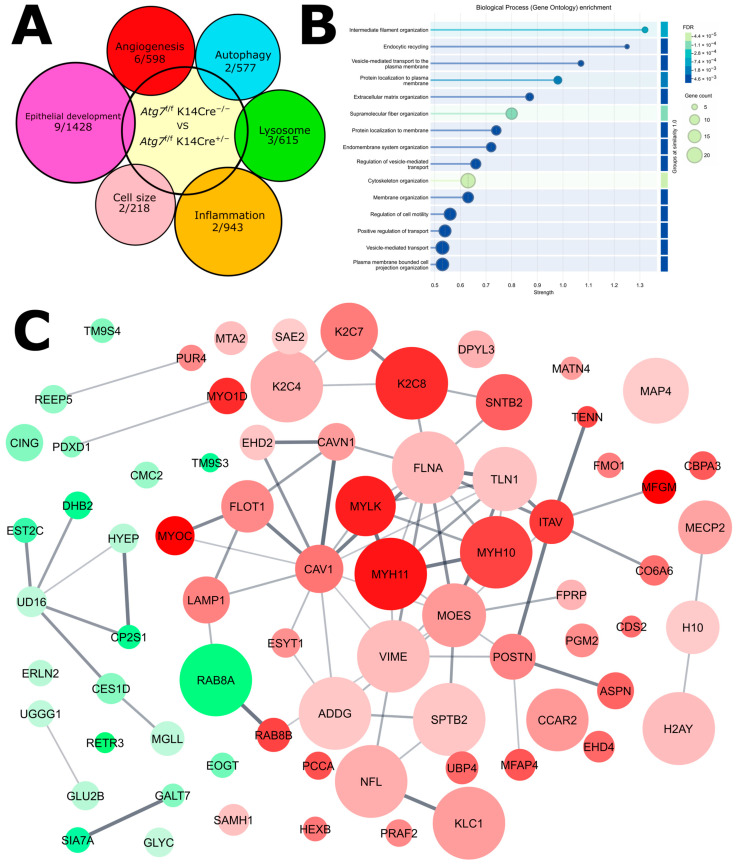
(**A**) Overlap between the proteins upregulated in and the corresponding genes involved in several pathways related to phenotypic characteristics observed in the ATG7-deficient mutant. Pathway genes were obtained from the GO database released on 22 July 2025. (**B**) GO Biological Process enrichment analysis for the genes related to the upregulated proteins in ATG7-deficient mice. (**C**) Protein–protein interaction network showing different proteins and the network they form, based on the STRING network 12.0, minimum interaction score of 0.400. Green color indicates upregulation and red color indicates downregulation, node size indicates involvement in cytoskeleton-assembly, and the thickness of the lines connecting the nodes indicates the number of interactions.

Proteomic analysis showed that ATG7 deficiency affects several cellular pathways (Figure 7A). Some of these changes are related to the observed increase in lymphatic sprouting and thickening of the epithelium. The most affected pathways involve filament organization, endocytic recycling, and vesicle transport (Figure 7B). These are important for autophagy and the movement of autophagy-related components. Protein–protein interaction analysis (Figure 7C) shows that increased and decreased proteins belong to separate pathways, except for RAB8A, which is involved in both.

## 3. Discussion

Autophagy is a cellular process that maintains corneal homeostasis by removing damaged organelles and proteins, thereby preserving cellular function and viability under both normal and stress conditions [30].

The *Atg7*^f/f^ K14Cre^+/−^ mouse model has been investigated in several tissue compartments, often showing clear phenotypes resulting from dysregulated autophagy. The cornea, a tissue that had not been investigated, showed distinct and functionally significant changes when ATG7 was removed. Our study demonstrates that ATG7 deficiency in the mouse corneal epithelium results in distinct alterations in autophagy markers, immune cell presence, and lymphatic vessel morphology without gross vascular remodeling. The observed increase in LC3B expression, accompanied by a reduction in LC3B-positive puncta in the basal epithelium, supports a blockade of autophagosome formation, consistent with ATG7’s essential role in LC3 lipidation [31]. The observed increase in LC3B signal, despite the reduction in puncta, may reflect cytosolic accumulation of non-lipidated LC3B (LC3-I) due to disrupted autophagic flux. These findings align with previous reports of autophagy impairment in ATG7 knockout models and suggest a spatially distinct autophagy phenotype in the corneal epithelium [8,31,32,33].

This is further confirmed by the absence of autophagosome-like structures in TEM images. This absence is consistent with the established role of ATG7 in the lipidation of LC3 and the elongation of autophagosomal membranes. The lack of autophagosomes at the ultrastructural level further confirms that autophagy is blocked upstream of vesicle formation in the ATG7-deficient cornea. These findings align closely with previous reports of impaired autophagy in ATG7-deficient tissues, which show disrupted autophagic flux and accumulation of non-lipidated LC3 [8].

Nevertheless, in previous research, there were neither obvious morphological differences nor clear functional defects observed in the cornea of autophagy-deficient animals, but an increased corneocyte thickness in dorsal skin was noted [10]. Here, we did observe a corneal phenotype relevant to epithelial morphology: increased epithelial thickness, specifically in the basal epithelium. This observation concurs with prior studies using the same K14-driven ATG7-deficient mouse model, which reported no overt effects in skin morphology apart from the increased corneocyte thickness [10]. These findings suggest that, despite profound molecular alterations, compensatory mechanisms may preserve gross tissue structure and function in the cornea under steady-state conditions.

A potential limitation of this model is the use of the K14Cre driver, which may cause mosaic and patch-like modifications in the corneal epithelium, as shown in previous publications [34,35]. The incomplete loss of ATG7 protein, which we observe may be a result of this mosaicism. To further investigate the role of ATG7, or to investigate earlier developmental relevance of ATG7, future studies could use Cre drivers active in earlier development, such as Le-Cre driven by the Pax6 P0 promoter [36,37].

Given autophagy’s well-established role in regulating immune responses and maintaining tissue homeostasis, it is likely that impaired autophagic clearance contributes to epithelial stress and subsequent immune activation. Similar associations have been reported in other models where autophagy disruption leads to enhanced inflammation and immune cell recruitment [38]. The localized increase in lymphatic vessel sprouts seen in the sutured animals despite unchanged overall vascular coverage, may reflect a lymphangiogenic response to an altered immune milieu, potentially facilitating immune cell trafficking within the cornea, as we confirm in our proteomic analysis.

Proteomic analysis revealed upregulation of RAB8, RETR3, and TM9S3. RAB8A is associated with secretory autophagy, sometimes termed exophagy, a process where vesicles are routed to the plasma membrane, where their content is expelled into the extracellular space [39]. RAB8A is the ubiquitously expressed isoform involved in vesicular transport, while the RAB8B isoform is rarer and is linked to developmental processes via Wnt target gene induction [34,35]. RETR3 is involved in endoplasmic reticulum–autophagosome binding, also termed reticulophagy, a process where selective autophagy takes place in the endoplasmic reticulum [36,37]. TM9S3 is a Golgi-resident transmembrane protein that binds to ATG8 and LC3 to regulate a form of Golgi-specific autophagy termed Golgiphagy [40,41,42]. Previous work on ATG7-deficient mice showed expansion of the endoplasmic reticulum and Golgi swelling [43]; this possibly explains the increased Golgi stress that could lead to Golgiphagy. The increased organelle swelling also supports the increased cell size we observed. The upregulation of these proteins suggests activation of ATG7-independent compensatory pathways, exophagy, reticulophagy, and Golgiphagy when the ATG7-dependent form of autophagy is disabled. Such adaptations have been observed in other autophagy-deficient tissues, underscoring the cellular plasticity in maintaining proteostasis despite impaired bulk autophagy [32]. Lysosomal marker LAMP1 is reduced in ATG7-deficient mouse cornea. This correlates with previous investigations that showed reduced LAMP1 accumulation in the white and brown adipose tissue of mice deficient for ATG7 in adipose tissue [44].

Cytoskeletal and vesicular proteins showed the most change in terms of affected proteins. There was a decrease in K2C4, K2C7, and K2C8, which form filament networks to give cells strength and shape [45]. Loss of Krt8 is also linked to hepatocyte “ballooning”, a dramatic increase in cell size [46] similar to what we observe in the cornea. A decrease in CAV1, a regulator of autophagic degradation under oxidative stress [47]. CAV1-deficient mice are in a low-grade pro-inflammatory state, particularly sensitive to infection [48,49]. The EHD proteins, EHD4 and FLOT1, are markers of vesicles and were reduced in the mutant animal [50,51]. Another EHD protein, EHD2, is a caveola mechanotransducer that can act as a transcription factor [52]; its absence suggests impaired mechanosensing. Apical extrusion components FLNA and VIME are also reduced in the ATG7-deficient mouse cornea; the absence of FLNA leads to impaired migration of daughter cells from the basal layer [53,54]. VIME and TLN1 are also components of the focal adhesion complex that regulates integrin-actin intracellularly [55]; the depletion of these components reduces the attachment of cells to the ECM and will likely affect cell morphology. MYO1D, a motor protein, facilitates vesicle movement along the cytoskeleton, an important part of autophagosome trafficking [56,57]. Spectrin–actin network components SPTB2 and ADDG both show reduced presence in the ATG7-deficient mice. This suggests a disruption of the spectrin–actin junction complex, a likely factor of the altered cell morphology we observed in the epithelium [58,59]. The coordinated downregulation of these proteins suggests that autophagy supports the maintenance of vesicle-trafficking systems necessary for its own function. CING quantity was increased, and overexpression of CING is linked to a reduction in cell contractility due to a decrease in myosin phosphorylation [60,61]. Combined with the loss of contractile cell components MYH10 and MYH11 that we observed [62,63], this supports the increased cell size we see in the basal epithelium.

The lymphatic sprout phenotype observed suggested a change in proteins that control the angiogenic and inflammatory processes. A decrease in MFGM was found; it is a secreted glycoprotein that promotes angiogenesis, and it is also a documented controller of autophagy in some cases [64,65,66]. There was also a reduction in integrin αV, an important component of vascular endothelial cell adhesion and migration. Integrin αV is an indirect promoter of autophagy via focal adhesion kinase, driving localized autophagy [67]. MECP2 also showed a decrease in the autophagy-deficient animal; this is a transcriptional regulator with a documented role as both a repressor and a requirement of autophagy in other tissues, as well as a role as a promoter of anti-inflammatory pathways [68,69,70,71]. MECP2 depletion suggests a dysregulation of autophagy and anti-inflammatory regulation. In the ATG7-deficient cornea, the downregulated angiogenesis-linked proteins are mostly tied to the regulation of autophagy, suggesting a shift in the autophagic program in addition to a shift in the inflammatory profile. The increase in proteins linked to alternative pathways such as exophagy, reticulophagy, and Golgiphagy supports this concept of autophagic redundancy. Previous work showed that knocking out Eph2a in the mouse cornea, which also inhibited autophagic flux, caused thickening of the corneal epithelium [72]. MGLL is an enzyme that directly regulates the levels of fatty acids [73]. Loss of MGLL in the oesophageal epithelium is associated with a pro-inflammatory phenotype [74].

Overall, proteomic analysis revealed that several pathways were affected by ATG7-deficiency. Some of these pathways can be linked to our observed phenotypes of increased lymphatic sprouting and thickened epithelium. The most affected pathways are filament organization, endocytic recycling, and vesicle transport. These correspond to autophagy and the process that supports the transport of the autophagic components. Organized as a protein–protein interaction network, it is clear that the depleted protein and increased proteins are part of separate pathways, with the exception of RAB8A.

As the first publication characterizing the ATG7 cornea, a broad, exploratory screen of protein presence has proved informative. A more in-depth analysis of relevant mechanistic pathways and the particular targets outlined here could follow up and give a better understanding of the underlying processes. Additionally, transcriptomic analysis, including siRNA targeting, would also round out the characterization of the molecular implications of the ATG7-deficiency in the cornea.

Our data characterized ATG7 loss in the corneal epithelium, with particular focus on autophagic dysfunction and subtle vascular remodeling. These findings highlight the importance of autophagy in corneal homeostasis and immune privilege, providing new insights into potential mechanisms underlying corneal inflammation and lymphangiogenesis. Future work should explore the functional impact of these changes on corneal transparency and wound healing, as well as investigate therapeutic strategies to restore autophagic balance in ocular surface diseases.

Clinically, the increase in lymphatic vessel sprouting observed in autophagy-deficient corneas may have important implications for ocular surface diseases. Lymphangiogenesis is a key contributor to corneal inflammation, transplant rejection, and impaired wound healing [28]. The increased lymphangiogenic sprouting may change outcomes in surgical interventions on the cornea, potentially exacerbating or prolonging inflammatory responses. Understanding this relationship opens potential avenues for therapeutic interventions aimed at modulating autophagy to control pathological lymphangiogenesis and improve outcomes in corneal inflammatory disorders [75].

## 4. Materials and Methods

### 4.1. Mice

All harvesting of animal tissue was approved by the Landesamt für Verbraucherschutz und Ernährung Nordrhein-Westfalen (LAVE) and conducted in strict adherence to the guidelines listed by the Association for Research in Vision and Ophthalmology Statement for the Use of Animals in Ophthalmic and Vision Research. The mice were housed under standard conditions with a 12:12 h light–dark cycle and ad libitum access to food and water.

A material transfer agreement was established with Dr. Masaaki Komatsu to perform experiments with *Atg7*^f/f^ mice [8]. The mice were sourced from previously crossed K14Cre mice (strain Tg (KRT14-cre)1 Amc/J) that produced *Atg7*^f/f^ and *Atg7*^f/f^ K14Cre^+/−^ [10,76]. The mice were in the C57BL/6J background. The animals used were between the ages of 10 and 12 weeks. The mice were sacrificed via either cervical dislocation or CO_2_ inhalation.

K14Cre transgenic mice express Cre recombinase under the control of the keratin-14 promoter, which is active in basal epithelial cells during late embryonic stages and postnatally. In the cornea, K14Cre-mediated recombination is known to be mosaic, leading to incomplete deletion of floxed alleles. This mosaicism must be taken into account when interpreting phenotypes in *Atg7*^f/f^ K14Cre^+/−^ mice.

### 4.2. RNA Extraction and RT-PCR Analysis

The RNA was extracted from the whole cornea and corneal epithelium with an RNeasy minikit (Qiagen, Venlo, The Netherlands) per the manufacturer instructions. Total RNA was quantified using the NanoDrop 2000c (Thermo Scientific, Waltham, MA, USA).

The cDNA was synthesized from 5 μg of DNase I-treated total RNA using the RevertAid First Strand cDNA Synthesis Kit (Thermo Scientific, USA).

The qPCR analysis was performed using the SsoFast EvaGreen Supermix (BioRad, Hercules, CA, USA), a CFX96 Real-time system (BioRad), and normalized to the reference gene transcript of HPRT.

Specific primers for each gene were as follows: ATG7 (Forward: TCC GTT GAA GTC CTC; Reverse: CCA CTG AGG TTC ACC), LC3B (Forward: CAG CAC CGA AAT GAT C; Reverse: CAC CAG AAT TGG CAG), HPRT (Forward: GTT GGA TAC AGG CCA GAC TTT GTT G; Reverse: GAT TCA ACT TGC GCT CAT CTT AGGC).

### 4.3. Western Blotting

The epithelium was separated from the cornea by digesting the mouse cornea in 20 mM EDTA for 20 min at 37 °C. The corneal epithelium was carefully peeled using forceps and a crescent knife. The epithelium was then submerged in 100 µL of RIPA Lysis and Extraction Buffer (Thermo Scientific, USA) at 4 °C. The epithelium was homogenized with a probe sonicator (Bandelin Sonopuls HD 2070, Bandelin electronic GmbH & Co. KG, Berlin, Germany) and centrifuged (14,000× *g*, 15 min, 4 °C). The supernatant was transferred to a new tube, and total protein content was quantified using a Pierce BCA protein assay kit (Thermo Scientific, USA). Protein extracts (1.5 mg/mL) were separated using 10% SDS-PAGE gel and transferred to a polyvinylidene fluoride (PVDF) membrane. Immunoblotting was performed using anti-ATG7 (A2856, Sigma Aldrich, St. Louis, MO, USA) used for immunostaining, an anti-Histone-H3 antibody (housekeeping protein) (17168-1-AP, Proteintech, Rosemont, IL, USA), and a mouse or rabbit secondary antibody coupled to horseradish peroxidase (DAKO, Waldbronn, Germany). The membranes were developed using an enhanced chemiluminescence reagent (Bio-Rad, Munich, Germany), and the images were captured using a Bio-Rad Molecular Imager^®^ Gel Doc™ XR System. Semi-quantification was carried out using ImageJ v1.54m. Signals were normalized against beta actin and the control group (0 mg/mL Bevacizumab).

### 4.4. Transmission Electron Microscopy

Mouse corneas were fixed with 2% GA/2% PFA in 0.1 M Cacodylate buffer for 48 h at 4 °C. The samples were then washed with 0.1 M Cacodylate buffer and stored in the same buffer until ready for post-fixation.

The relevant region of the cornea was immersion-fixed in 2% formaldehyde (Science Services, München, Germany #E15711), 2% glutaraldehyde (Sigma, St. Louis, MO, USA # G5882-100ML), in 0.1 M sodium cacodylate buffer (Applichem, Darmstadt, Germany #2140,0250) for 48 h at 4 °C. Samples were washed with 0.1 M sodium cacodylate buffer. Post-fixation was applied using 2% OsO4 (Science Services, München, Germany # E19190) in 0.1 M cacodylate buffer for 2 h at 4 °C. Samples were washed four times with 0.1 M Cacodylate buffer and dehydrated using an ascending ethanol series (50%, 70%, 90%, 3 × 100%) for 15 min each. After incubation with a mix of 50% (*v*/*v*) ethanol/propylenoxide and two times with pure propylenoxide for 15 min each step, samples were infiltrated with a mixture of 50% (*v*/*v*) epon/propylenoxide for 2 h and 70% (*v*/*v*) epon/propylenoxide for 2 h each at 4 °C. Then the samples were transferred into fresh pure epon (Science Services, München, Germany # E14120) and let them incubate overnight at 4 °C. The next day, epon was exchanged and samples were incubated for 2 h at RT, placed into PELCO 21-cavity embedding mold (Plano, Marburg, Germany 10505) and cured for 72 h at 60 °C.

Ultrathin cross sections of 70 nm were cut using an ultramicrotome (Leica Microsystems, Wetzlar, Germany UC6) and a 45° diamond knife (Diatome, Nidau, Switzerland). Sections were stained with 1.5% uranyl acetate (Agar Scientific, Rotherham, UK # R1260A) for 15 min at 37 °C and with 3% Reynolds lead citrate solution made from Lead (II) nitrate (Roth, Karlsruhe, Germany # HN32.1) and tri-Sodium citrate dehydrate (Roth, Karlsruhe, Germany #4088.3) for 4 min. Images were acquired using a JEM-2100 Plus Transmission Electron Microscope (JEOL, Freising, Germany) operating at 80 kV equipped with a OneView 4 K camera (Gatan, Pleasanton, CA, USA).

### 4.5. Proteomics

#### 4.5.1. Labeling for SP3

For quantitative proteomics, cell pellets were resuspended in 5% SDS in 1× PBS. Nucleic acids were digested using Benzonase HC (25 units per 5 × 10^5^ cells) (Sigma, St. Louis, MO, USA). Dithiothreitol (DTT) was added to a final concentration of 5 mM, and samples were incubated at 55 °C for 30 min. Chloroacetamide (CAA) was then added to a final concentration of 40 mM, followed by a 30 min incubation at room temperature in the dark. Protein concentration was estimated using A280 measurements on a Nanodrop 2000c. Two treatment groups were selected for analysis: Cre-positive mouse corneas and Cre-negative mouse corneas.

Samples were analyzed using a Q Exactive Exploris 480 mass spectrometer (Thermo Scientific) equipped with a FAIMSpro ion mobility device, coupled to an UltiMate 3000 HPLC system (Thermo Scientific). Peptides were first loaded onto a 5 µm PepMap trap cartridge precolumn and then reverse-flushed onto a custom-packed analytical column (30 cm × 75 µm I.D.) containing 2.7 µm Poroshell EC120 C18 resin (Agilent, Santa Clara, CA, USA). Chromatographic separation was performed at a constant flow rate of 300 nL/min using the following gradient: 2% to 6% solvent B (0.1% formic acid in 80% acetonitrile) over 1 min, then to 32% over 72 min, 55% over the next 7 min, and finally to 95% over 2 min. A 6 min wash at 95% solvent B followed. The FAIMSpro device operated at a compensation voltage of −50 V, with electrode temperatures set to 99.5 °C (inner) and 85 °C (outer).

#### 4.5.2. Quantitation

MS1 scans were acquired over a range of 390 to 1010 *m*/*z* at a resolution of 15,000, with a maximum injection time of 22 ms and an AGC target of 100%. MS2 scans covered 300 to 1500 *m*/*z*, also at 15,000 resolution, with the same injection time and AGC settings. Data-independent acquisition (DIA) was performed across the 400 to 1000 *m*/*z* range using 75 staggered windows of 8 *m*/*z*, resulting in 150 nominal 4 *m*/*z* windows after demultiplexing. All scans were recorded in centroid mode.

#### 4.5.3. Data Processing

Thermo raw files were demultiplexed and converted to mzML format using the msconvert module in ProteoWizard. A human canonical SwissProt FASTA file (downloaded on 26 June 2020) was processed into a Prosit-compatible input file using the convert tool in EncyclopeDIA v0.9.0 [77] with default settings: Trypsin digestion, up to one missed cleavage, *m*/*z* range 396–1004, charge states 2+ and 3+, default charge state 3+, and normalized collision energy (NCE) of 33. The resulting CSV file was uploaded to the Prosit web server and converted into a spectral library in generic text format [78]. This library, containing 20,374 protein isoforms, 28,307 protein groups, and 1,626,266 precursors, was used with DIA-NN v1.7.16 to generate a sample-specific library using the match-between-runs (MBR) function. Key settings included: 0.01 FDR filter, N-terminal methionine excision enabled, maximum one missed cleavage, minimum peptide length of 7, maximum peptide length of 30, precursor *m*/*z* range 400–1000, cysteine carbamidomethylation set as a fixed modification, and double-pass search enabled.

### 4.6. Immune Cell and Vessel Imaging

Corneas were collected to quantify blood vessel and lymphatic vessel coverage, as well as CD45+ leukocyte count. The corneas were fixed in acetone for 30 min, washed with PBS, and then blocked with 2% BSA in PBS. The corneas were flattened with four cuts, and excess materials such as muscle or tissue were cut away. These flattened corneas were stained with CD31 to visualize blood vessels and with LYVE-1 to identify lymphatic vessels. CD45+ leukocytes were visualized with an anti-CD45 antibody.

Quantification of vessels was performed using Cell^F^ software version 3.4 (Build 2677) (Olympus, Münster, Germany). Grayscale images of the stained cornea were processed using specific filters. The innermost vessel of the limbal arcade was used to identify the corneal edges. Threshold analysis was then applied to determine the area occupied by vessels. The percentage of each vessel type was calculated relative to the total area of the cornea.

Quantification of CD45+ leukocytes was performed using FIJI software v1.54m and the specific plugins Trainable Weka Segmentation [79] and Labkit [80] to identify CD45+ cells in the cornea. The individual CD45+ cells in the central cornea were counted.

### 4.7. ATG7 Stain and LC3B Puncta Quantification

Eyes were collected to observe LC3B activity in the corneal epithelium. To make sections, the whole eye was mounted in OCT compound (Sakura Finetek, St. Torrance, CA, USA) and cryo-sectioned. The sections were fixed in 4% PFA for 10 min, washed with PBS, and then blocked with 2% BSA in PBS. The sections were stained with either antibody: anti-ATG7 (A2856, Sigma Aldrich, St. Louis, MO, USA) or anti-LC3B (ab192890, Abcam, Cambridge, UK). DAPI and a secondary anti-rabbit a647 antibody (A21245, Invitrogen, Carlsbad, CA, USA) were used. When LC3B localizes to autophagosomes, it appears as punctate structures. When LC3B is not recruited to autophagosomes, it appears diffuse in the cytoplasm. The LC3B puncta were counted in the cells of the central corneal basal epithelium.

### 4.8. Corneal Suture Model

The corneal suture model was performed as previously described [81]. Three figure-eight sutures made with 11/0 monofilament polyamide suture (Serag-Wiessner, Naila, Germany) were set into the corneal stroma of 10-week-old mice for 2 weeks. Animal welfare was monitored throughout the 2 weeks. The animals were sacrificed after the two-week period, and the corneas were collected for vessel staining and imaging.

### 4.9. Statistical Analysis and Graphing

Statistical analysis was carried out with PRISM 10.4.0 software (GraphPad, San Diego, CA, USA). Student’s *t*-test with Mann–Whitney post-test was used. A *p*-value lower than 0.05 was defined as statistically significant. Results were graphed with GraphPad PRISM, MaxQuant Perseus 4.1.3.0 [82], SRPlot version April 2025 [83], STRING 12.0 [84], and Cytoscape 3.10.3 [85].

## 5. Conclusions

Our study reveals that ATG7 deficiency in the corneal epithelium results in marked autophagic impairment, immune activation, and subtle lymphatic remodeling without overt changes in overall corneal morphology. The disruption of autophagosome formation, confirmed by molecular and ultrastructural analyses, underscores the indispensable role of ATG7 in autophagic flux. The increased presence of immune cells and lymphatic sprouts highlights how autophagy supports immune regulation and vascular quiescence in the cornea. Proteomic evidence of compensatory mechanisms, including alternative modes of autophagy and membrane trafficking, indicates cellular adaptation to ATG7 loss. These insights emphasize autophagy’s multifaceted contribution to corneal integrity and immune privilege, suggesting that therapeutic modulation of autophagy may hold promise for treating corneal inflammatory and degenerative conditions. Future research should focus on how these molecular and cellular changes affect corneal transparency, wound healing, and long-term tissue function.

## Data Availability

Publicly available proteomics datasets were analyzed in this study. The mass spectrometry proteomics data have been deposited in the ProteomeXchange Consortium via the PRIDE [86] partner repository with the dataset identifier PXD068453 (accessed on 17 September 2025).
